# From adversity to adaptation: the struggle between resilience and athlete burnout in stressful situations

**DOI:** 10.3389/fpsyg.2025.1578198

**Published:** 2025-05-30

**Authors:** Chenyi Cai, Zhengyang Mei, Yang Yang, Shi Luo

**Affiliations:** School of Physical Education, Southwest University, Chongqing, China

**Keywords:** athlete, resilience, burnout, neurobiological mechanisms, theoretical models

## Abstract

Athlete burnout is considered a negative psychological consequence of stressors in sports, with the potential to adversely affect both the physical and mental well-being of athletes, as well as their overall performance. Resilience serves as a protective factor against stressors, enabling athletes to effectively manage the unique challenges they encounter in the sports environment, ultimately helping protect them from burnout. This narrative review aimed to summarize the direct evidence regarding the relationship between resilience and athlete burnout. By examining existing theories and empirical evidence, the relationship between resilience and athlete burnout was explored and discussed in terms of individual and environmental factors, theoretical models, and neurobiological mechanisms to construct effective intervention programs to prevent and control the occurrence of athlete burnout. At present, resilience can positively influence athlete burnout through individual factors (perceived stress, coping strategies, and motivation) as well as environmental factors (social support, motivational climate, family cohesion, and coach-athlete relationship). The theoretical models mainly include the stress coping process model of athlete resilience and the systematic self-reflection model. These models elucidate the relationship between resilience and athlete burnout from the viewpoints of stress coping and self-reflection, respectively. The neurobiological mechanisms through which resilience influences athlete burnout are explored primarily through the hypothalamic–pituitary–adrenal (HPA) axis and the mesolimbic dopamine system. These mechanisms suggest that resilience has an effect on athlete burnout primarily through stress hormone levels and brain region activity related to reward and motivation. However, the pathways and mechanisms through which resilience influences athlete burnout require further in-depth investigation. Future research should combine the longitudinal perspective to track the dynamic impact relationship between resilience and athlete burnout and focus on examining the relevant theoretical framework from a multidisciplinary research perspective to provide a theoretical basis for coaches and sports managers to formulate scientific training programs. This will help enhance athlete resilience, effectively prevent and mitigate the risk of burnout, and promote the development of athletes’ physical and mental well-being.

## 1 Introduction

Athlete burnout refers to the immediate and transient physical and psychological stress responses experienced by athletes, resulting from a significant imbalance between their individual resources and situational demands (Raedeke, 1997). When athletes fail to recover effectively from prolonged stress, the stress response accumulates and eventually evolves into a state of physical and psychological symptoms characterized by emotional/physical exhaustion, reduced athletic accomplishment and sport devaluation (Raedeke, 1997). Specifically, emotional/physical exhaustion stems from the psychosocial and physical demands associated with high-intensity training and competition; reduced athletic accomplishment refers to a loss of efficacy and tends to negatively assess sports performance and ability; and sport devaluation is a kind of negative and detached attitude toward sports, manifested primarily by a lack of concern for performance (Raedeke, 1997; Raedeke et al., 2002). Over the past two decades, the average incidence of athlete burnout has progressively increased, encompassing a range of negative psychological states and behavioral patterns emerged during training and competition. These include decreased motivation for sport, impaired competitive performance, heightened perceived stress (De Francisco et al., 2016; Goodger et al., 2007; Gustafsson et al., 2008; Isoard-Gautheur et al., 2016), and even leading to premature retirement (Gustafsson et al., 2017). Evidence has suggested that stress can serve as a predictor of burnout, with burnout being a negative consequence of stress (De Francisco et al., 2016; Moen et al., 2015). Prolonged exposure to stressful situations can result in chronic stress, which may cause athletes to lose motivation for continued participation in sports (Tabei et al., 2012). Given the adverse impact of burnout on athletes, it is crucial to prevent and alleviate burnout by fostering resilience.

Resilience refers to an individual’s ability to cope and recover effectively in the face of setbacks and adversity and maintain normal physiological function and psychological health (Gupta and McCarthy, 2022; Mei et al., 2024). As a protective factor linked to stress, resilience is directly related to athletes’ subjective assessment of stress events and their choice of coping strategies (Fletcher and Sarkar, 2013). The sporting resilience meta-model suggests that a lack of resilience to maintain the balance between individual resources and situational demands may ultimately result in physical and psychological dysfunction, thereby increasing the risk of burnout (Richardson et al., 1990). Research indicates that resilience plays a crucial role in fostering athletes’ positive coping in stressful situations, which in turn prevents athlete burnout (Galli and Vealey, 2008). However, the specific mechanisms through which resilience influences athlete burnout remain unclear, limiting a comprehensive understanding of the underlying connection between the two. In light of this, this study aimed to provide a narrative review of direct evidence regarding the relationship between resilience and athlete burnout, and to summarize its research findings. By examining existing theories and empirical evidence, the relationship between resilience and athlete burnout was explored and discussed in terms of individual and environmental factors, theoretical models, and neurobiological mechanisms to construct effective intervention programs to prevent and control the occurrence of athlete burnout. On the basis of these analyses, research prospects are proposed to expand the understanding of how resilience influences athletes’ psychological states and behavioral patterns, providing a theoretical reference and guidance for subsequent research in this field.

## 2 Methods

We conducted a literature search via electronic databases, including PubMed, Web of Science, and Google Scholar. In addition, we referred to other studies with similar study designs and identified potential studies meeting the inclusion criteria from their reference lists. The search period ranged from the inception of the database to February 2025. The keyword combinations used in the search strategies consisted of (“‘Resilience” OR “Resilien*”) AND (“Athlet*” OR “Elite*” OR “Sport*”) AND (“Burnout*”). In terms of the inclusion criteria, in addition to the literature being published in English and peer reviewed, it must also meet at least one of the following conditions: (a) Examining individual or environmental factors through which resilience influences athlete burnout; (b) Examining theoretical models of how resilience influences athlete burnout; or (c) Examining the neurobiological mechanisms through which resilience influences athlete burnout. The first and second authors reviewed the retrieved literature to assess whether it aligns with the narrative scope of this study. The quality of the included studies was assessed via mixed-methods appraisal tool (MMAT) (Hong et al., 2018).

## 3 Results

The electronic databases yielded 311 records, with 31 records from PubMed, 137 records from Web of Science, and the remaining 143 records from Google Scholar and reference lists. After 38 duplicate records were removed, the titles, abstracts, and full texts of the remaining records were screened, and 273 records were identified and eliminated because they fell outside the narrative scope of this study. A total of 10 studies were ultimately included in the review (Lu et al., 2016; Malán-Ernst et al., 2025; Raanes et al., 2019; Santana et al., 2023; Sorkkila et al., 2019; Tutte-Vallarino et al., 2022; Vitali et al., 2015; Wagstaff et al., 2018; Wu et al., 2022; Yıldırım et al., 2023). In terms of study characteristics, the included studies were published between 2015 and 2025 and involved 3,302 athletes from various sports. These studies were conducted in multiple countries, including Uruguay, China, Norway, Mexico, Finland, Italy, the UK, and Turkey. Nine studies used a cross-sectional design and one study used a longitudinal design.

Lu et al. (2016) employed a cross-sectional research design, involving 218 athletes as participants. Resilience and burnout were assessed using the abbreviated version of the Connor-Davidson Resilience Scale-2 and the Athlete Burnout Questionnaire, with questionnaires distributed offline before or after the training sessions of each team. The results indicated that resilience and coaches’ social support conjunctively moderated the stress-burnout relationship.

Malán-Ernst et al. (2025) employed a cross-sectional research design, involving 194 athletes as participants. Resilience and burnout were assessed using the adapted version of the Resilience Scale and the Inventory of Burnout in Athletes Revised, with questionnaires distributed offline before the season. The results indicated that resilience could serve as a protective factor to reduce the risk of burnout in high performance athletes.

Raanes et al. (2019) employed a cross-sectional research design, involving 670 athletes as participants. Resilience and burnout were assessed using the Resilience Scale for Adults and the Athlete Burnout Questionnaire, with questionnaires distributed online over a four-week period. The results indicated that resilience was negatively associated with burnout symptoms in young athletes.

Santana et al. (2023) employed a cross-sectional research design, involving 308 athletes as participants. Resilience and burnout were assessed using the Spanish Version of the Resilience Scale and the Athlete Burnout Questionnaire, with questionnaires distributed offline. The results indicated that resilience appeared to be a protective factor against the development of athlete burnout.

Sorkkila et al. (2019) employed a longitudinal research design, involving 491 athletes as participants. Resilience and burnout were assessed using the Sport Burnout Inventory-Dual Career Form and the Brief Resilience Scale, with questionnaires distributed offline at the beginning of the first academic year, six months after the end of the first academic year, one year after the end of the second academic year, and 6 months after the start of the third academic year. The results indicated that resilience could prevent student-athletes from burning out and dropping out from sport and school.

Tutte-Vallarino et al. (2022) employed a cross-sectional research design, involving 121 athletes as participants. Resilience and burnout were assessed using the Resilience Scale and the Inventory of Burnout in Athletes Revised, with questionnaires distributed offline after the season. The results indicated that resilience obtained a clear influence on the occurrence of burnout.

Vitali et al. (2015) employed a cross-sectional research design, involving 87 athletes as participants. Resilience and burnout were assessed using the revised Resilience Scale and the Athlete Burnout Questionnaire, with questionnaires distributed offline before the start of regular training sessions. The results indicated that resilience had a protective effect on athlete burnout.

Wagstaff et al. (2018) employed a cross-sectional research design, involving 372 athletes as participants. Resilience and burnout were assessed using the Connor-Davidson Resilience Scale and the Athlete Burnout Questionnaire, with questionnaires distributed through a combination of online and offline methods. The results indicated that resilience was a salient individual difference variable that buffers against potential negative outcomes.

Wu et al. (2022) employed a cross-sectional research design, involving 506 athletes as participants. Resilience and burnout were assessed using the Connor-Davidson Resilience Scale and the Athlete Burnout Questionnaire, with questionnaires distributed online during the season. The results indicated that for athletes with high resilience, the indirect effect of organizational stressors on athlete burnout via competitive trait anxiety was weaker.

Yıldırım et al. (2023) employed a cross-sectional research design, involving 335 athletes as participants. Resilience and burnout were assessed using the Brief Resilience Scale and the Athlete Burnout Questionnaire, with questionnaires distributed online. The results indicated that the adverse impact of fear of failure on athlete burnout can be mitigated by cultivating resilience. The main characteristics, quality appraisals, and research findings of the included studies are presented in Tables 1, 2.

**Table 1:** Main characteristics and quality appraisal of the included studies.

Study ID	Country	Study design	Sample size	Event	Quality appraisal					
					Rating	Methodological quality criteria				
Lu et al. (2016)	China	Cross-sectional study	218 athletes	Multi-sport	****	N	Y	Y	Y	Y
Malán-Ernst et al. (2025)	Uruguay	Cross-sectional study	194 athletes	Multi-sport	****	N	Y	Y	Y	Y
Raanes et al. (2019)	Norway	Cross-sectional study	670 athletes	Multi-sport	***	N	Y	Y	N	Y
Santana et al. (2023)	Mexico	Cross-sectional study	308 athletes	Multi-sport	***	N	Y	Y	Y	N
Sorkkila et al. (2019)	Finland	Longitudinal study	491 athletes	Multi-sport	***	N	Y	Y	N	Y
Tutte-Vallarino et al. (2022)	Uruguay	Cross-sectional study	121 athletes	Multi-sport	*****	Y	Y	Y	Y	Y
Vitali et al. (2015)	Italy	Cross-sectional study	87 athletes	Multi-sport	****	N	Y	Y	Y	Y
Wagstaff et al. (2018)	UK	Cross-sectional study	372 athletes	Multi-sport	****	N	Y	Y	Y	Y
Wu et al. (2022)	China	Cross-sectional study	506 athletes	Multi-sport	***	N	Y	Y	N	Y
Yıldırım et al. (2023)	Turkey	Cross-sectional study	335 athletes	Multi-sport	****	N	Y	Y	Y	Y

Y, Yes; N, No.

**Table 2:** Research findings of the included studies.

Study ID	Research findings
Lu et al. (2016)	Resilience and coaches’ social support conjunctively moderated the stress-burnout relationship
Malán-Ernst et al. (2025)	Resilience could serve as a protective factor to reduce the risk of burnout in high performance athletes
Raanes et al. (2019)	Resilience was negatively associated with burnout symptoms in young athletes
Santana et al. (2023)	Resilience appeared to be a protective factor against the development of athlete burnout
Sorkkila et al. (2019)	Resilience could prevent student-athletes from burning out and dropping out from sport and school
Tutte-Vallarino et al. (2022)	Resilience obtained a clear influence on the occurrence of burnout
Vitali et al. (2015)	Resilience had a protective effect on athlete burnout
Wagstaff et al. (2018)	Resilience was a salient individual difference variable that buffers against potential negative outcomes
Wu et al. (2022)	For athletes with high resilience, the indirect effect of organizational stressors on athlete burnout via competitive trait anxiety was weaker
Yıldırım et al. (2023)	The adverse impact of fear of failure on athlete burnout can be mitigated by cultivating resilience

## 4 Discussion

### 4.1 Correlational research on the impact of resilience on athlete burnout

According to the resilience model proposed by Richardson et al. (1990), resilience plays a crucial role not only in helping individuals develop more effective coping strategies for life difficulties but also in enhancing their ability to withstand adversity. In the context of athlete burnout, resilience exerts its influence through a mediating effect that connects individual and environmental factors. For example, athlete with higher resilience exhibit higher levels of positive adaptability, socialization, communication, and problem-solving skills in the face of stress (Özdemir, 2019). Additionally, they possess a clear goal orientation and maintain an optimistic view of the future, which makes them less susceptible to the negative impact of stressors (Dugdale et al., 2002; Martin-Krumm et al., 2003; Vitali, 2012). Therefore, the correlational research on the impact of resilience on athlete burnout has focuses on individual and environmental factors.

#### 4.1.1 Individual factors

Achieving excellence and competitive success in sports is challenging for athletes, as intense training from an early age is needed to sufficiently prepare them for the competitive season (Gould and Whitley, 2009). However, prolonged years of intense training and competition mean that such groups are inevitably subjected to individual or organizational stressors (Fletcher et al., 2008; Manzi et al., 2010). According to the general stress theory, athlete burnout is regarded as a negative outcome resulting from prolonged and continuous exposure to stressors (Hobfoll and Freedy, 2017; Lin et al., 2022). Numerous empirical studies support this theory, demonstrating a significant positive correlation between athlete burnout and perceived stress (Cohn, 1990; De Francisco et al., 2016; Gustafsson and Skoog, 2012; Raanes et al., 2019). The sporting resilience meta-model suggests that in the face of stressors, resilience can buffer or mitigate the negative impact of stressors on athletes’ physical and mental well-being (Gupta and McCarthy, 2022). A cross-sectional study based on student-athletes confirmed that athletes with high resilience are less prone to experience maladaptive outcomes from stress and that resilience moderates the relationship between stress and burnout in athletes (Lu et al., 2016). Similarly, a cross-sectional study examining the relationship between organizational stressors and athlete burnout indicated that while high levels of perceived stress increase the likelihood of burnout symptoms, the moderating effect of resilience assists athletes in managing and reducing stress, ultimately decreasing their burnout levels (Wu et al., 2022). Specifically, athletes with low resilience tend to perceive stressors as threats and experience more frequent worry, physiological tension, and impaired attention, which ultimately increases their risk of burnout (Wu et al., 2022). In contrast, athletes with high resilience are more likely to view stressors as challenges and opportunities for career growth. This positive motivation for sports helps them to proactively overcome and cope with stressful situations, thereby reducing the risk of burnout. In summary, resilience acts as both a “filter” and a “buffer” between stressors and athlete burnout, shielding athletes from the negative impact of stressful situations and thus reducing the risk of developing maladaptive psychological states and behavioral patterns.

The stress-emotion-performance meta model proposed by Fletcher and Sarkar (2013) suggests that resilience can influence the choice of coping strategies during athletes’ exposure to stressors, resulting in either adaptive or maladaptive coping outcomes (Fletcher and Scott, 2010). Athletes with high resilience tend to develop a range of effective strategies to actively mobilize their resources in response to adversity and stress, thereby effectively alleviating associated negative emotions (e.g., burnout) (Kim and Duda, 2003; Secades et al., 2016). Research has confirmed a significant positive correlation between resilience and adaptive coping strategies but a significant negative correlation with maladaptive coping strategies (Secades et al., 2016). Specifically, athletes with high resilience tend to adopt adaptive coping strategies, such as problem-oriented coping, threat minimization, or seeking social support (Yi et al., 2005). Conversely, athletes with low resilience tend to adopt maladaptive strategies, such as avoidance-oriented coping, blaming others, or wishful thinking (Yi et al., 2005). Athletes with high resilience actively seek solutions to problems in the face of stress and in turn, experience higher levels of self-efficacy and optimism, ultimately reducing burnout symptoms (Xiaomei et al., 2024). In conclusion, resilience helps athletes maintain an optimistic mindset and stay focused on problem-solving when confronted with stress, promotes the adoption of adaptive coping strategies, reduces the generation of negative emotions, and thus reduces the risk of burnout.

The self-determination theory suggests that individuals have three basic psychological needs: autonomy, competence, and relatedness. These needs play crucial roles in motivational behavior, and there are different correlation patterns with psychological states and behavioral patterns (Deci and Ryan, 2013). For athletes, prolonged setbacks or failure to meet basic psychological needs during their careers can lead to a lack of intrinsic motivation, thereby increasing the risk of developing burnout symptoms (Cresswell and Eklund, 2006; Cresswell and Eklund, 2005). Several studies have confirmed a significant negative correlation between intrinsic motivation and athlete burnout, whereas external motivation or a lack of motivation is significantly positively correlated with athlete burnout (Cresswell and Eklund, 2005; Gould et al., 1996; Raedeke and Smith, 2001; Yıldırım et al., 2023). The motivated effort-allocation model of self-regulation points out that individuals with greater internal motivation are more inclined to allocate their own psychological resources (e.g., resilience) to reduce stress-induced maladaptive coping outcomes (e.g., athlete burnout, competition anxiety) (Molden et al., 2016). A cross-sectional study examining the relationship between resilience and motivation revealed that resilience positively influences athletes’ intrinsic regulation (Nascimento Junior et al., 2021). Specifically, athletes with high resilience have a greater capacity to independently manage and overcome stress, which increases their intrinsic motivation for regular training and competition, thereby reducing maladaptive stress responses (e.g., burnout). A cross-sectional study based on elite athletes confirmed that for those with low resilience, external motivation tends to dominate their training and competition, with goals being typically driven by rewards or external factors, which in turn increases burnout and decreases their satisfaction (Yıldırım et al., 2023). However, few existing empirical studies have directly explored how resilience moderates the relationship between motivation and athlete burnout, implying that the validity and generalizability of this evidence need further investigation to deepen the understanding of the specific associative patterns between resilience and athlete burnout.

In summary, previous research has identified various individual factors, each examining how resilience influences athlete burnout through different mediating variables. A substantial body of evidence demonstrates that resilience improves the ability to recover from adversity and stress, both at the individual and environmental levels, thereby reducing susceptibility to burnout (Lu et al., 2016; Yi et al., 2005). Therefore, future research could expand in the following aspects: (a) a comprehensive inclusion of various individual factors, such as self-efficacy, focus, confidence, and self-esteem, is necessary to explore the specific conditions under which resilience mediates athlete burnout, which would help refine the pathways through which resilience influences burnout and inform the development of more targeted intervention strategies from multiple perspectives; (b) building on the findings of the cross-sectional study, a longitudinal study design should be implemented to track athletes over time, which would allow for a more systematic and dynamic examination of the impact mechanisms of resilience and burnout; and (c) developing and implementing highly targeted resilience interventions, including mental skills training (Birrer and Morgan, 2010), mindfulness training (Haase et al., 2015), gratitude interventions (Gabana et al., 2022), and stress training interventions (Kegelaers et al., 2021), would allow for the evaluation of the impact of different resilience interventions on athlete burnout.

#### 4.1.2 Environmental factors

In stressful situations, the onset of athlete burnout must consider the the interaction between the individual and the environment. Based on the grounded theory of psychological resilience and optimal sport performance, perceived social support, as an important environmental factor related to resilience, can buffer or protect athletes from the potential negative impact of stressors (e.g., burnout) (Fletcher and Sarkar, 2012; Galli and Vealey, 2008). Previous studies have reported a significant positive correlation between resilience and athletes’ perceived social support (Pedro and Veloso, 2018; Sullivan et al., 2023; Yi et al., 2005). Athletes with high resilience are likely to have well-developed interpersonal skills, enabling frequent interactions with coaches, teammates, and family members throughout training sessions and competitions. These consistent interactions facilitate their ability to recognize and effectively receive social support. The stress-buffering hypothesis suggests that high levels of perceived social support protect an individual from the potential negative impact of stressors (e.g., athlete burnout) (Cohen and Wills, 1985). Multiple studies have confirmed a significant negative correlation between perceived social support and athlete burnout (Cresswell, 2009; Raedeke and Smith, 2001, 2004; Shang and Yang, 2021). Lu et al. (2016) explored the relationship between perceived social support and burnout levels based on student-athletes and the results reported that perceived social support enables athletes to mobilize more physical and psychological resources in stressful situations, thus helping them resist the negative impact related to stress. Similarly, a study based on Brazilian athletes confirmed that perceived social support not only helps athletes maintain focus during training and competition but also effectively enhances their ability to cope with organizational stressors, thereby mitigating the adverse psychological consequences of stressors (e.g., burnout) (Codonhato et al., 2018). In addition, perceived social support provides athletes with crucial resources to manage burnout, which can help alleviate physical and psychological exhaustion, mitigate feelings of reduced accomplishment, offer advice for enhancing performance, and encourage the growth of healthy sport motivation, ultimately lowering the level of athlete burnout (Pacewicz et al., 2019). In summary, owing to their perception of greater social support, athletes with high resilience are likely to exhibit greater resistance in the face of adversity and stress, potentially preventing burnout more effectively.

The resilience process theory contends that resilience is characterized by dynamic interactions between individuals and their environment (Egeland et al., 1993). In the context of sports, this interplay can impact athletes’ perceptions of the motivational climate. Research has shown that resilience can moderate the impact of perceived motivational climate on burnout symptoms to some extent (Vitali et al., 2015). The perceived motivational climate includes both the perceived performance motivation (ego-involving) climate and the perceptive mastery motivation (task-involving) climate, which are associated with various psychological outcomes (e.g., athlete burnout) (Ames, 1992; Nicholls, 1984). Vitali (2012) reported a significant relationship between athlete resilience and the motivational climate among male athletes. Specifically, athletes with high resilience are more likely to adopt mastery-oriented motivation, seeking success through personal effort in training and competition. In contrast, athletes with low resilience tend to focus on performance-oriented motivation, deriving a sense of achievement and satisfaction from comparing themselves to others, which increases their susceptibility to burnout. Multiple studies have confirmed a significant positive correlation between athletes’ perceived performance motivation climate and burnout, whereas a significant negative correlation exists between the perceived mastery motivation climate and athlete burnout (Appleton and Duda, 2016; Lemyre et al., 2008; Reinboth and Duda, 2004; Vitali et al., 2015). Furthermore, athletes’ perceived mastery motivation climate can stimulate their desire for challenges and increase intrinsic interest and motivation, thus protecting them from burnout. Conversely, athletes’ perceived performance motivation climate tends to focus on the validation of self-worth and the perception of inadequate ability, which in turn heightens feelings of threat and anxiety and may ultimately lead to burnout symptoms in athletes (Lemyre et al., 2008). Given this, resilience plays an important role in influencing the type of motivational climate perceived by athletes, and further influences the relationship between the perceived motivation climate and athlete burnout. To promote the physical and mental well-being of athletes in sports activities, coaches should strive to create a task-involving motivational climate while avoiding the reinforcement of an ego-involving motivational climate.

### 4.2 Theoretical models of the impact of resilience on athlete burnout

#### 4.2.1 Stress coping process model of athlete resilience

**Figure 1:**
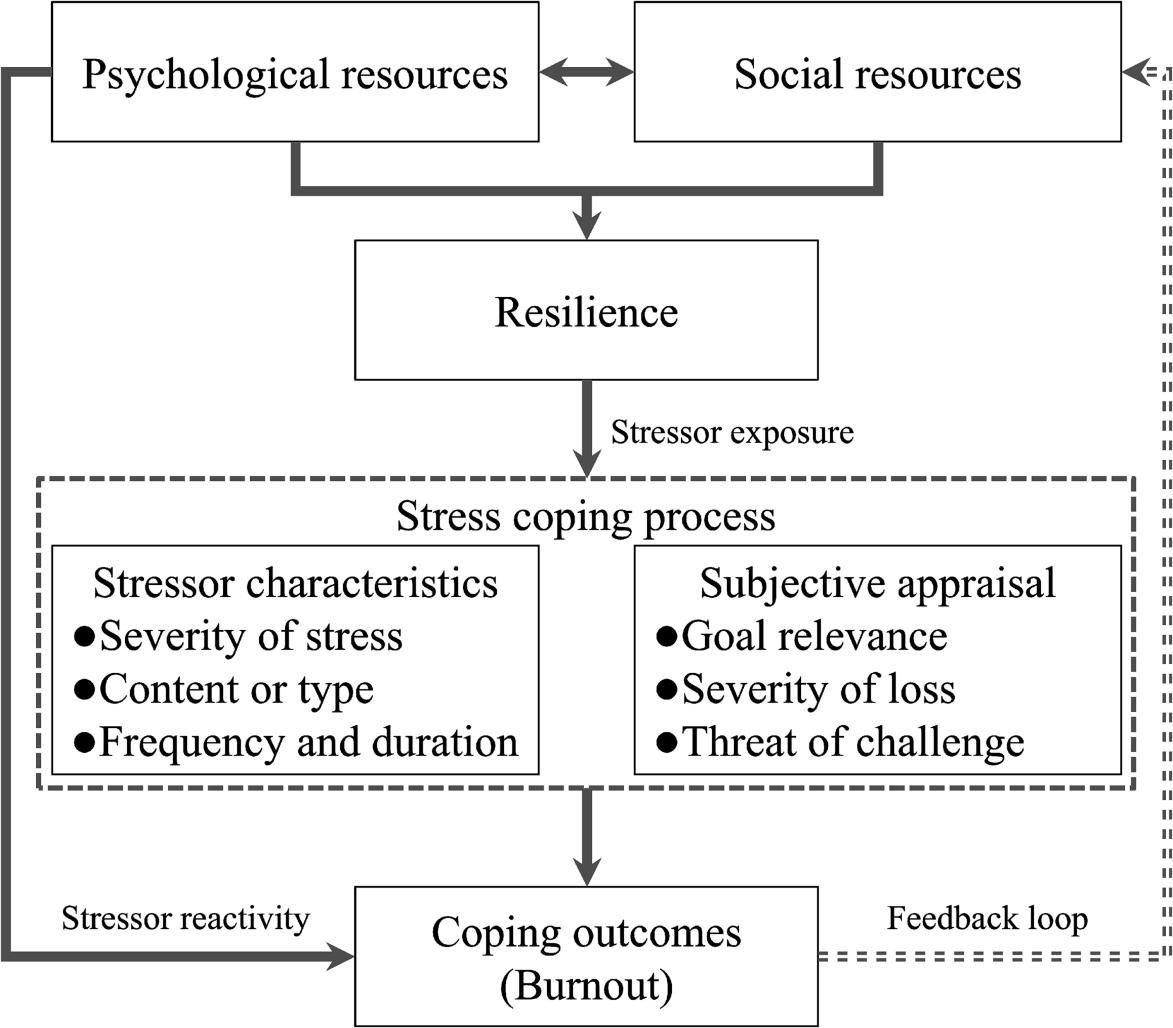
Stress coping process model of athlete resilience.

Based on the stress coping process model of athlete resilience (see Figure 1), athlete resilience levels are determined primarily by psychosocial resources that influence the relationship between stressor exposure and stress coping process, potentially leading to a series of psychological states and behavioral patterns associated with stress coping (Almeida, 2005). The stress coping process is composed of both stressor characteristics (severity of stress, content or type, and frequency and duration) and athletes’ subjective appraisal of these stressors (goal relevance, severity of loss, and threat of challenge). This process is affected by stressor exposure and psychosocial resources related to athlete resilience (Almeida, 2005). Furthermore, psychosocial resources can also have a direct effect on athletes’ stress coping outcomes through stressor response (the possibility of athletes’ emotional or physical reactions to stressors), and stress coping outcomes may in turn have a feedback effect on psychosocial resources, thus affecting athletes’ physical and mental well-being (Almeida, 2005; Diehl et al., 2012). This feedback effect indicates that successful coping may enhance athlete resilience, whereas coping failure may impede the development of athlete resilience (Diehl et al., 2012). The stress coping process model of athlete resilience aligns with the social ecology of resilience, where stressors and psychosocial resources jointly determine the athletes’ stress coping process and the negative outcomes they experience (Ungar, 2011).

Research has confirmed that the psychosocial resources available to athletes allow them to positively evaluate and interpret the relationship between stressors and their own resources, helping them cope with and adapt to stress and ultimately reducing negative coping outcomes such as burnout (Fletcher and Sarkar, 2012; Fletcher and Sarkar, 2013). Additionally, athlete resilience levels are influenced by their stress coping experiences and outcomes, which supports the feedback effect proposed by the stress coping process model (Graber et al., 2015; Richardson, 2002; Richardson et al., 1990). A qualitative study based on elite athletes suggested that their stressful experiences will inspire their confidence to overcome adversity and stress in the future (Morgan et al., 2013). This is because athletes’ accumulated experiences from negative events can mobilize previously unused psychosocial resources, which helps bolster their resilience and thus more effectively enables them to cope with and adapt to future stressful situations (Seery, 2011; Seery et al., 2010). On the basis of this model, when faced with stress from training or competition, athletes with high resilience tend to subjectively assess stressors in a more positive way, which helps maintain physical and mental health, thereby reducing the risk of burnout symptoms.

In summary, the stress coping process model of athlete resilience explains the relationship between athlete resilience and burnout from the perspectives of psychosocial resources, stressors and subjective evaluation, which fully reflects the interaction between individual and environmental factors in the stress process. However, this model still has several limitations that need to be addressed. Specifically, although the model indicates that athlete resilience can influence their subjective evaluation of stressors, the precise process and mechanisms of its influence have not been clearly explained. Similarly, how negative coping outcomes related to stressors affect athlete resilience through feedback pathways also needs to be further explained to reveal the possible bidirectional relationship from a more comprehensive and dynamic perspective. In light of this, additional empirical research grounded in the stress coping process model is necessary to provide more reliable evidence, fill the gaps in current models, and further enhance the understanding of how resilience fosters athletes’ physical and mental well-being.

#### 4.2.2 Systematic self-reflection model

**Figure 2:**
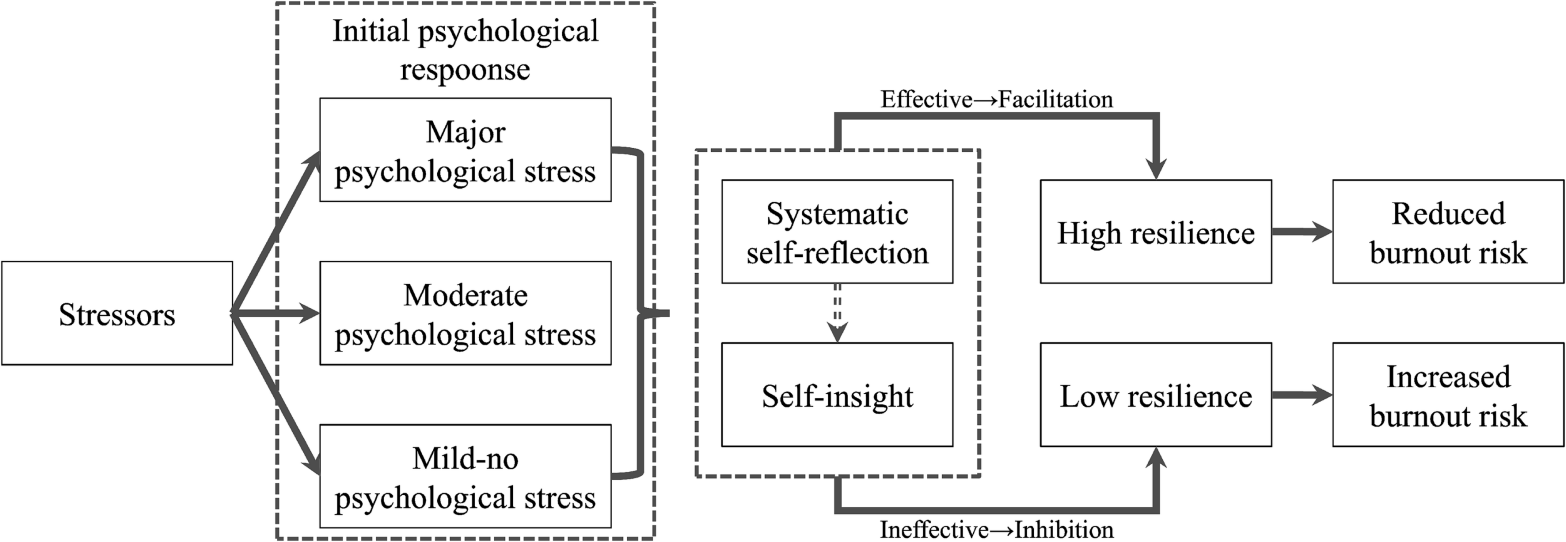
Systematic self-reflection model.

Self-reflection is defined as a meta-cognitive method of learning that involves the development of self-awareness and the assessment of one’s thoughts, feelings, and behaviors (Grant et al., 2002; Hattie et al., 1996). The systematic self-reflection model suggests that when individuals encounter stressors, self-reflection can aid in coping with these challenges, thereby enhancing resilience and reducing the maladaptive impact of stressors on the individual (see Figure 2) (Crane et al., 2019). Specifically, the model views individuals’ initial psychological response to stressors as a prerequisite for triggering the self-reflection process. Self-reflection helps individuals develop adaptive coping strategies (task-oriented and problem-solving), thereby enhancing resilience and enabling more effective coping with stressful situations (Crane et al., 2019). The initial psychological response to stressors are categorized into three levels: major, moderate, and mild to no psychological stress, with self-reflection being more likely to occur at the moderate level of psychological stress, which is most effective in counteracting maladaptive coping outcomes associated with stressors (e.g., athlete burnout) (Crane et al., 2019). In contrast, too few or too many psychological stress may fail to effectively trigger athletes’ self-reflection and resilience, resulting in a diminished role of self-reflection and resilience in this process (Crane et al., 2019). This is because the relationship between the degree of stress exposure and resilience follows an inverted U-shaped curve, that is, appropriate stress exposure is associated with high resilience (Seery et al., 2010). The systematic self-reflection model is similar to the cognitive-motivational-relational theory proposed by Lazarus (2000), which states that when athletes face stressful situations, they can engage in adaptive self-reflection, view stressful events as challenges and respond to them positively to buffer the negative impact of stressors on their physical and mental state and thus reduce the possibility of burnout.

Studies have confirmed that athletes’ self-reflection is a significantly positively correlated with resilience that and self-reflection can negatively predict adverse psychological outcomes related to stressors, such as psychological stress and pre-competition anxiety (Cowden and Meyer-Weitz, 2016; Wang, 2023). Given this, athletes with strong self-reflection may have better emotional control and self-confidence when confronted with stressful situations, which can help mobilize more resilience resources to cope with adversity and stress, and thus reduce the risk of burnout. Furthermore, the process through which athletes enhance resilience to cope with adversity and stress via self-reflection also encompasses self-insight, which may serve as a mediator in the relationship between the two (Bucknell et al., 2022). Compared with self-reflection, self-insight plays a more crucial role in enhancing resilience and mitigating maladaptive coping strategies linked to stress (Cowden and Meyer-Weitz, 2016). In stressful situations, athletes with high levels of self-insight are more inclined to seek and utilize available external resources while also developing positive cognitive flexibility (Cowden and Meyer-Weitz, 2016). By clarifying and understanding their thoughts and feelings, athletes may more effectively cope with situational demands and adjust their psychological and behavioral responses to overcome adversity and stress, thereby being less prone to burnout symptoms (Cowden and Meyer-Weitz, 2016).

From the perspective of the systematic self-reflection model, in stressful situations, athletes’ self-reflection contributes to strengthening their resilience, thereby effectively preventing maladaptive outcomes (e.g., burnout) associated with stressors. However, most empirical studies on the systemic self-reflection model are cross-sectional, failing to uncover or fully illustrate the causal relationships proposed by the model. Therefore, it is essential to investigate the specific mechanisms linking resilience, burnout, and self-reflection from a longitudinal perspective, thereby clarifying the impact relationship between resilience and self-reflection to provide empirical support for reducing athlete burnout. Additionally, during training and competition, coaches and relevant sports administrators should implement appropriate interventions to foster athletes’ self-reflection. These interventions could include psychological skills training, encouraging athletes to maintain training logs, and creating a supportive environment. This would help reduce the influence of negative external factors and enable athletes to better withstand the negative impact of stressors on athletes’ physical and mental well-being.

### 4.3 Neurobiological mechanisms of the impact of resilience on athlete burnout

The neurobiological mechanisms through which resilience influences athlete burnout are explained primarily based on the hypothalamic–pituitary–adrenal (HPA) axis and the brain reward systems. Specifically, the HPA axis is a crucial endocrine stress system in the human body, with cortisol being its primary end product and a key hormone in stress response (Lupien et al., 2007). Individuals experiencing chronic stress lead to overactivation of the HPA axis, which in turn increases cortisol levels, and cortisol has a negative feedback effect on the brain (mainly through glucocorticoids), which affects the remodeling of the limbic system of the brain (Yang et al., 2011), which may increase the an individual’s susceptibility to burnout. An integrated model of athlete burnout states that elevated cortisol levels are an early sign of burnout, which can subsequently result in negative outcomes, including weakened immune function and withdrawal or exit from the sport (Gustafsson et al., 2011). In the field of sports, the stress generated by training and competition significantly elevates athletes’ cortisol levels, leading to lower resilience, which further hinders their adaptive coping with stress, thereby accelerating the development of burnout (Filaire et al., 2009; Raanes et al., 2019). Athletes with low resilience typically have increased levels of perceived stress (Hjemdal et al., 2006; Sarkar, 2017), increasing their vulnerability to the adverse impact of stressors, which may impair the top-down regulatory function of the prefrontal cortex, leading to increased cortisol secretion and triggering negative affective responses (e.g., burnout). In contrast, athletes with high resilience are able to regulate cortisol secretion by assessing stressors, thereby mitigating maladaptive responses to stressors and contributing to enhanced sports performance (Gustafsson et al., 2011). Studies have confirmed that there is a significant negative correlation between total cortisol release in athletes and their resilience during training and competition and that resilience can modulate the potential negative impact of high cortisol levels on athletes’ psychological state and behavioral patterns (Filaire et al., 2009), thereby helping to reduce the risk of burnout.

In addition, the effect of resilience on athlete burnout may also be based on brain regions involved in reward and motivation regulation, such as the mesolimbic dopamine system. Dopamine in the mesolimbic dopamine system primarily regulates an individual’s motivation, pleasure experiences, and behavior through the ventral tegmental area and the nucleus accumbens (Berridge and Robinson, 1998; Humińska-Lisowska, 2024). Evidence shows that stress can influence dopamine levels and dopaminergic neuronal activity in the mesolimbic dopamine system, thereby enabling individuals to respond behaviorally to a variety of environmental stimuli (Baik, 2020). Stress can reduce an individual’s sensitivity to rewards, potentially leading to a loss of pleasurable experiences or a lack of motivation (Baik, 2020), characteristics that align with athlete burnout. Furthermore, the degree of stress exposure has an inverted U-shaped relationship with dopamine release, with mild and transient stressors having an activating effect on dopamine release, whereas intense, chronic and uncontrollable stress inhibits dopamine release (Cabib and Puglisi-Allegra, 2012; Marinelli, 2007). Therefore, during training and competition, reduced dopamine release may expose athletes to a greater risk of burnout, as they lack the motivation to train or compete, have difficulty experiencing positive emotions, and ultimately fail to meet their basic psychological needs. In contrast, increased resilience in athletes is associated with high dopamine levels (Humińska-Lisowska, 2024), which contribute to regulating the stress response, enabling athletes to stay focused and composed in stressful situations (Nowacka-Chmielewska et al., 2022; Willmore et al., 2022), thereby reducing the likelihood of negative stress coping outcomes will occur (e.g., burnout). However, direct evidence regarding the specific mechanisms through which dopamine influences athlete resilience is still lacking. One possible explanation is the multidimensional nature of stressors and their interaction with various neurotransmitter systems, not solely dopamine (Martín-Rodríguez et al., 2024). The dopamine system plays a key role in regulating athletes’ motivation and reward expectations, enabling them to sustain their coping abilities under stress and effectively overcome the challenges and difficulties encountered in competitive sports (Schultz, 2016). This is especially vital for athletes in building confidence, fostering success expectations, and reducing the risk of burnout during training and competition.

### 4.4 Implications for future research

On the basis of individual and environmental factors, theoretical models, and neurobiological mechanisms, the present study examined the impact of resilience on athlete burnout in stressful situations. First, according to the general stress theory, self-determination theory, grounded theory of psychological resilience and optimal sport performance, as well as resilience process theory, the restrictive impact of resilience on athlete burnout through different pathways has been extensively explored. Second, various theoretical models, mainly including the stress coping process model of athlete resilience and the systematic self-reflection model, have been employed to explain the impact of resilience on athlete burnout. Finally, the influence mechanism of resilience on athlete burnout has been elucidated from the neurobiological perspective, and it has been suggested that the HPA axis and the mesolimbic dopamine system may play dominant and decisive roles in activating the impact of resilience on athlete burnout. At present, although many valuable results have been obtained in research on the impact of resilience on athlete burnout, some issues that require further resolving in future research remain.

The pathways through which resilience influences athlete burnout, as well as potential mediators and moderators within this process, could be further explored and examined. The process through which resilience influences athlete burnout is bound to be influenced by many mediating variables. Current research has focused primarily on individual factors in athletes, including perceived stress, coping strategies, and sport motivation (Cresswell and Eklund, 2006; De Francisco et al., 2016; Secades et al., 2016). This implies that some socio-cultural factors specific to the sport environments have been ignored, such as coach autonomy support, team cohesion, coach-athlete relationships, and teammate relationships, which may facilitate or inhibit the influence of resilience on athlete burnout through potential meditating roles. In addition, some demographic factors, including gender, age, and region, may play a moderating role in the process through which resilience influences athlete burnout. For example, in stressful situations, male athletes may be more inclined than female athletes to distance themselves from negative thought patterns and develop problem-solving strategies, which may help reduce the likelihood of burnout. Therefore, it is necessary for future research to broaden the horizon, fully incorporate socio-cultural and demographic factors, and examine their potential limiting roles in the process of resilience affecting athlete burnout. This approach will offer a more comprehensive and accurate understanding of the complex relationship between resilience and burnout to provide a scientific basis for developing more effective intervention strategies.

By adopting a longitudinal research approach, it would be possible to track how resilience influences the relationship between stress and burnout over an extended period. Few previous studies have explored the impact of resilience on athlete burnout from a longitudinal research perspective, which has made it difficult to establish causal relationships between these variables. As a continuous and dynamic process, sports training exposes athletes to a range of stressful events at different levels, with a temporal dimension ranging from transient stress (e.g., loss of points during a game) to long-term stress (e.g., serious injury). Similarly, athlete resilience is a dynamic process, implying that the relationship between resilience and athlete burnout may vary across different training periods. Consequently, future research can examine the dynamic relationship between resilience and athlete burnout by constructing longitudinal samples of athletes at various time points (e.g., preseason) or in different contexts (e.g., during preparation for major competition). Furthermore, how resilience influences burnout through athletes’ challenge appraisal and meta-cognition during different training periods should be examined based on qualitative research findings. For example, implementing a longitudinal interview program would not only reduce the likelihood of recall bias but also allow for a detailed capture of the dynamic evolution of athletes’ thoughts, emotions, and behaviors as they cope with sports adversity. Employing these methods would enable the identification of additional protective factors linked to resilience while also analyzing their roles in relation to different stressors, which in turn provides scientific evidence for developing targeted psychological interventions that align with athletes’ actual needs.

Through empirical and intervention studies, the theoretical models could be further verified and developed, while incorporating multidisciplinary perspectives to enrich the theoretical framework regarding the impact of resilience on athlete burnout. The stress coping process model of athlete resilience and the systematic self-reflection model explain the inhibitory effect of resilience on athlete burnout through various pathways (Almeida, 2005; Crane et al., 2019). However, these models focus primarily on individual factors to explore the impact of resilience on athlete burnout, without adequately exploring the potential interactions between resilience and other relevant variables. In light of this, it is necessary to design more high-quality research to test the scientificity and validity of existing theoretical models and, on this basis, integrate multidimensional factors to improve their predictive and explanatory power. In addition, existing models are rooted mainly in psychology, suggesting that future research needs to draw on theories and methods from biology, neuroscience, cognitive science, social science, and other fields to construct an interdisciplinary model to understand the impact of resilience on athlete burnout from a multidimensional perspective.

Currently, the neurobiological mechanisms through which resilience influences athlete burnout mainly involve on the impact of brain reward areas on the secretion of cortisol and dopamine. However, it is necessary to further explore the role of other brain regions or structures in this impact relationship. Studies have shown that athlete burnout is similar to other symptoms induced by chronic stress (e.g., posttraumatic stress disorder, major depressive disorder, and chronic fatigue syndrome); that is, they are all are linked to dysfunction of the HPA axis (Jameson, 2016; Mommersteeg et al., 2007). On the basis of the current understanding of the neurobiological mechanisms through which resilience influences athlete burnout, future research could explore intervention experiments in the following areas: (a) by engaging in mental skills training, athletes can improve their self-efficacy and emotional regulation, enabling them to manage adversity and stress in a more constructive way; (b) integrated autonomic biofeedback training could be employed to enhance athletes’ self-regulation and stress-coping ability. This could involve monitoring indicators such as skin conductance level, blood pressure, and heart rate variability to observe biofeedback, improving perceived stress and burnout symptoms in athletes; and (c) functional magnetic resonance imaging could be employed to identify changes in the activity of the limbic system and prefrontal cortex in athletes during stressful situations, which could help in understanding the neurobiological mechanisms underlying stress and burnout, ultimately leading to the development of targeted interventions to improve athlete resilience and prevent burnout.

## 5 Conclusion

Although some studies provide direct evidence of the relationship between resilience and athlete burnout, the individual and environmental factors involved in the impact of resilience on athlete burnout still require further in-depth exploration. Relevant theoretical models and neurobiological mechanisms should be further refined based on existing research to expand insights into the role of resilience in improving athletes’ maladaptive psychological states and behavioral patterns.
